# Health-related quality of life and depression among medical sales representatives in Pakistan

**DOI:** 10.1186/s40064-016-2716-1

**Published:** 2016-07-11

**Authors:** Muhammad Atif, Arslan Bashir, Quratulain Saleem, Rabia Hussain, Shane Scahill, Zaheer-Ud-Din Babar

**Affiliations:** Department of Pharmacy, The Islamia University of Bahawalpur, Bahawalpur, Pakistan; Lahore Pharmacy College, Lahore Medical and Dental College, Lahore, Pakistan; School of Management, Massey University, Auckland, New Zealand; School of Pharmacy, University of Auckland, Auckland, New Zealand

## Abstract

Pharmaceutical companies have been known to pose stress and mental harassment on medical sales representatives (MSRs) in-order to increase pharmaceutical sales. This cross sectional descriptive study, conducted during November and December 2014 in the Lahore and Bahawalpur districts of Punjab, Pakistan, evaluates the Health-Related Quality of Life (HRQoL) and extent of depression among MSRs in Pakistan. The significant predictors of HRQoL and depression among the MSRs were also determined. Using a convenience sampling technique, all consenting MSRs (N = 318) of pharmaceutical companies were asked to self-complete the Short Form-36 (SF-36v2) Health Survey and Stanford Personal Health Questionnaire (PHQ-8). The standard scoring scheme for the SF36v2 and PHQ-8 questionnaires was used. The PHQ-8 scores showed that 16.4 % (n = 52) and 2.5 % of respondents were suffering from major depression and severe major depression, respectively. Being depressed and having difficulty in achieving sales targets were the factors independently associated with lower physical health. Similarly, depression, insufficient time for the family and monthly income less than 36,000 Pakistan Rupees were significant predictors of lower mental health. The factors associated with depression included insufficient time for the family and unsatisfactory behavior of the managers. Compromised mental health and the prevalence of depression among the MSRs suggest pharmaceutical companies need to devise health management strategies and interventions to ensure effective prevention and management of mental health problems among Pakistani MSRs.

**Electronic supplementary material:**

The online version of this article (doi:10.1186/s40064-016-2716-1) contains supplementary material, which is available to authorized users.

## Background

Depression is of great concern for people working in all walks of life and so is their quality of life. It is a disease that results in sadness, low level of interest, self-worth and concentration. Feelings of tiredness and guilt along with compromised sleep and appetite are also the attributes of depression (World Health Organization 2016). Another important construct that is related to depression is Health-Related Quality of Life (HRQoL), which refers to the impact of perceived health on an individual’s ability to live a fulfilling life (Bullinger et al. 1993). There lies a complex inverse relationship between depression and HRQoL, where a surge of depression leads to poor HRQoL (Moore et al. 2005; Daly et al. 2010; Sim et al. 2005). It has been described that socio-demographically deprived and depressed patients are at a greater risk of poor HRQoL (Daly et al. 2010). The comfort and ease of employees at work are among the major challenges of the twenty-first century. Dissatisfied, frustrated, oppressed and exhausted employees become the victims of depression due to unachievable job targets, annoying working environments and insufficient salaries (Rafiq et al. 2012).

The value of the global Pharmaceutical market is growing and was approximately 650 billion United States Dollars (USD) in 2008–2009 and 1.1 trillion USD in 2014. The share of Japan, European Union and the United States of America (USA) is 12, 28 and 48 % respectively, with 20 % being contributed by the remaining countries of the world (Zaman 2011). The pharmaceutical market in Pakistan is about 21.59 billion Pakistan Rupees (PKR) with a growth rate of 16.4 %. The number of pharmaceutical firms registered with the Ministry of Health (MoH) in Pakistan is 600. Approximately 80 % of the local market requirement is met by some 400 pharmaceutical firms which include 28 multinational firms. There are approximately 40,000 registered brands of medicines with 1400 molecules marketed in Pakistan. Value wise, the market share of the pharmaceutical industry in Pakistan is 55–60 % (Ahmed et al. 2014).

Employing medical sales representatives (MSRs) is the most extensively used marketing technique by pharmaceutical firms. The MSRs act as a link between the healthcare professionals and pharmaceutical firms. Selling of prescription and nonprescription drugs and medical equipment, by creating awareness among the healthcare professionals as to the uniqueness of their product, persuading them to prescribe it, are some basic duties of the MSRs. In western countries, the pharmaceutical market is captured by positioning a single sales representative for every eight general practitioners (Caudill et al. 1996). However, the number of MSRs varies in different companies in Pakistan. A medium sized company has about 500 MSRs, and the number increases with the size of the pharmaceutical firm. There are approximately 25,000 MSRs in Pakistan, each having a call list of about 200 physicians with a target of visiting approximately 120 physicians every 4–6 weeks (Ahmed et al. 2014). Working at different geographical locations with specialty groups, organizing group discussions along with face to face meetings with doctors are the core elements of the job description of the MSRs in Pakistan (Kumar and Panigrahi 2014). However, most of the time they use *‘other’* techniques to promote the sale of pharmaceuticals. For example, offering gifts to the doctors, paying for their recreational and conference visits, offering monetary benefit on per unit sales of a pharmaceutical and so forth. This is because of unhealthy competition among the pharmaceutical firms in Pakistan. It is seen that the stressful working conditions and the irrational nature of the sales targets assigned to the MSRs hinder their productivity at work by causing depression, which results in their diminished HRQoL, ultimately reducing the work outcome on their part and the organization’s progress from a broader perspective (Behrman and Perreault 1984). Studies from India (Patil and Meena 2013), Japan (Sakakibara et al. 2006), the United Kingdom (UK) (Sang et al. 2010) and Turkey (Tander et al. 2007) have reported musculoskeletal problems and stress among MSRs in those countries.

In Pakistan, no satisfactory work has been undertaken by the regulatory authorities or independent researchers to highlight the challenges faced by the MSRs. Therefore, the present study was conducted to gauge the HRQoL, assess the level of depression and to determine the significant predictors of HRQoL and depression among MSRs in Pakistan. This study is expected to contribute in several ways. First, it plays a pivotal role in formulating labor rights, protecting regulatory authorities in Pakistan. Second, it helps to understand the level of depression and impairment in HRQoL of MSRs in Pakistan. Finally, it informs strategies to reduce the harm posed to the health of the MSRs in Pakistan.

## Methods

### Study setting and population

The present study was conducted among the MSRs promoting the sale of pharmaceuticals at various private and public healthcare facilities in the districts of Bahawalpur and Lahore, Punjab, Pakistan.

Lahore is the capital city of the Punjab province of Pakistan and is the second largest metropolitan area in the country and an important historical center in South Asia. It is considered to be one of the 35 largest cities of the world. An estimate in July 2014 put the population of the Lahore agglomeration at 7,566,000. There are about 14 public and 35 private hospitals in Lahore. Beside this, many doctors have private practices in different parts of the Lahore district. According to a rough estimate, 1500–2000 MSRs market their products in Lahore. We collected data from the MSRs at their contact points (place where MSRs meet with their managers/colleagues to discuss their work schedule) around the public and private sector hospitals. Public sector hospitals included; Jinnah Hospital, Sheikh Zaid Hospital, Meu Hospital and Punjab Institute of Cardiology. Private sector hospitals included; Omer Hospital, Mid City Hospital, Raza Medical Complex, Shoukat Khanam Hospital, Inmol Hospital and Surgimed Hospital. Besides this, we also met MSRs outside private clinics in the vicinity of the selected hospitals.

Bahawalpur is located in the southern part of the Punjab province of Pakistan. It is the 12th largest city of Pakistan with an approximate population of 3,333,467. The city was once the capital of the former princely state and later the province of Bahawalpur. There are three public sector tertiary care facilities in Bahawalpur. There are approximately, 500–750 MSRs representing various pharmaceutical companies in Bahawalpur district. Many doctors independently practice in their private clinics. We collected data from the MSRs at their contact points around Bahawal Victoria Hospital and various private clinics.

### Survey instrument

The survey instrument comprised of three parts. The first part aimed to collect the information about the demographics and other characteristics of the respondents. The second and third parts were the Short Form-36 version 2 (SF-36v2; HRQoL assessment tool) Health Survey and Stanford Personal Health Questionnaire-8 (PHQ-8; depression assessment tool), respectively.

The demographics of the respondents included: qualifications, job title, availability of quarterly shopping cards, annual leave allowances, professional training, periphery visits, the level of sales targets assigned, the nature of managerial and doctor’s behaviour, job experience, job security, number of calls made per day, product sample availability, the management of call timings, specialty group and the provision of pension/gratuity or provident fund by the company.

#### SF-36v2 Health Survey and its scoring

The SF-36 Health Survey is a generic HRQoL measuring tool which was first developed in the USA (Ware et al. 1998). In 1996, a new and improved version of the SF-36 called the ‘international version’ (i.e., SF-36v2) was developed which was considered to be superior owing to its qualities of better cultural adaptation, amended instructions, revised questions with diminished doubts and biases in the wording and a better ability to be translated. SF-36v2 is an extensively used, reliable and valid tool to measure the HRQoL in both diseased and disease free populations (Turner-Bowker et al. 2013).

SF-36v2 has eight scales that gauge eight domains of HRQoL listed as; Physical Functioning (PF, 10 items), Role-Physical (RP, four items), Role-Emotion (RE, three items), Bodily Pain (BP, two items), Vitality (VT, four items), Social Functioning (SF, two items), General Health (GH, five items) and Mental Health (MH, five items). Eight health domains are further summarized into two; Physical Component Summary (PCS) and Mental Component Summary (MCS) (Ware et al. 2007). The PF, RP and BP scales strongly correlate with the PCS, while the MH, RE, and SF scales strongly correlate with the MCS (Wang et al. 2008). The GH and VT moderately correlate with physical and mental components (Ware et al. 2007). Studies have shown that the PCS and MCS scores are easier to interpret and simpler to analyze statistically (McHorney et al. 1993; Ware et al. 2007).

Based on the recommendations of the developers and Asian studies (Ware et al. 2007; Atif et al. 2013), the standard scoring algorithms (United States weights) were used to obtain the standard norm-based scores (NBS) of eight health domains and the summary components. The Quality Metric’s QM Certified Scoring Software (version 4.5) was used to score the questionnaires.

The scores on health domain scales and component summary measures (PCS and MCS) ranging from 47 to 53 represented an average health level. Whereas, a score on a health domain scale or component summary measure less than 47 was considered indicative of impaired function within that health domain or dimension (Ware et al. 2007).

Permission to use the questionnaire was obtained from QualityMetric Incorporated. The validated Urdu (National language of Pakistan) version of the SF-36v2 Health Survey was provided by QualityMetric Incorporated, Lincoln, USA. The internal consistency of the majority of the health domains for the Urdu version of SF36v2 was within the admissible range, except for the VT (α = .66) and SF (α = .52) scales.

#### *PHQ*-*8 and its scoring*

PHQ-8 is widely used to assess the depression level in a given population [6]. It has eight questions with minimum and maximum possible scores of 0 & 24, respectively. The scoring assumptions of PHQ-8 are; <10 = no clinical depression, 10–19 = major depression and ≥20 severe major depression. Major depression and severe major depression were further categorized into clinical depression.

Using the standard translation methodology (forward and backward translation), the PHQ-8 was translated into Urdu. Before administering the translated questionnaire, it was pilot tested through involving 10 % of the target population. The internal consistency of the PHQ-8 questionnaire was .89.

### Study design and data collection

This was a cross-sectional and descriptive study. Using a convenience sampling technique, the MSRs were met at their contact points (meeting points) in the public and private hospitals and private clinics of BWP and Lahore during morning, afternoon, evening and night time according to their work schedule. Well-trained data collectors explained the purpose of the study to the target population and respondents who agreed to participate were asked to self-complete the SF-36v2 and PHQ-8 questionnaires. Participation in the study was voluntary. Demographic and other characteristics of the respondents were obtained by interviewing the respondents. All respondents signed written informed consent. Before completing the questionnaires, the participants were encouraged to read the ‘respondent information pack’. The permission to conduct the study was obtained from the Department of Pharmacy, the Islamia University of Bahawalpur, Pakistan.

### Statistical analysis

The data were analyzed using the PASW (Predictive Analysis Software, version 19.0. Armonk, NY: IBM Corp.). All continuous variables were reported as mean (SD) whereas; the categorical variables were described using counts and proportions (%). Simple linear regression analysis was used to examine the possible association between HRQoL scores (i.e., PCS and MCS scores) and selected socio-demographic and clinical variables. Only the statistically significant variables in the univariate analysis were entered into a multiple linear regression analysis to predict the final determinants of HRQoL. Beta, 95 % confidence interval (CI) for beta, standard error and *p* value were reported for each variable. Similarly, logistic regression analysis was used to determine the independent factors associated with clinical depression (major depression and severe major depression). The variables which were statistically significant (i.e., p-value <0.05) in the univariate analysis were entered into a multiple logistic regression analysis to predict the final independent factors. The adjusted odd ratios (AOR), 95 % confidence interval (CI), beta, standard error and p-value were reported for each predictor. The model fit was assessed by Chi square, degrees of freedom and p-value. Pseudo R square values were reported to provide information about the percentage of variance explained by the regression models. A p-value of <0.05 was considered statistically significant. The internal consistency of SF-36v2 and PHQ-8 were assessed using Cronbach’s alpha coefficient (Santos 1999; Pallant 2013). An alpha value equal to or greater than .70 was considered acceptable (Alhabahba et al. 2006).

## Results

### Characteristics of the respondents

A total of 440 respondents were approached. Out of these, 318 agreed to participate yielding a response rate of 72.3 %. 309 (97.2 %) participants were male. A majority of the consented respondents i.e. 230 (72.3 %) were between the age groups of 25 and 34 years. A total of 52 (16.4 %) respondents had pharmacy degrees. Unsatisfactory behaviour of the doctors and pharmaceutical company managers was reported by 33 (10.4 %) and 21 (6.6 %) participants, respectively. A detailed description of the characteristics of the respondents is given in Table 1.

**Table 1:** Characteristics of the respondents (N = 318)

Characteristics	Frequency (%)	Characteristics	Frequency (%)
Gender		Quarterly shopping cards	
Male	309 (97.2)	Yes	28 (8.8)
Female	9 (2.8)	No	290 (91.2)
Age group (years)		Annual leave	
18–24	44 (13.8)	Yes	257 (80.8)
25–34	230 (72.3)	No	61 (19.2)
35–44	41 (12.9)	Periphery visits	
45–54	3 (0.9)	Yes	234 (73.6)
Marital status		No	84 (26.4)
Single	174 (54.7)	Professional trainings	
Married	106 (33.3)	Yes	280 (88.1)
Married with children	36 (11.3)	No	38 (11.9)
Divorced/separated	2 (0.6)	Annual sales conference	
Title of last degree		Yes	277 (87.1)
B.Sc.	73 (23.0)	No	41 (12.9)
B.A./B.com.	133 (41.8)	Sales target assigned	
Masters	34 (10.7)	Yes	299 (94.0)
Pharmacy	52 (16.4)	No	19 (6.0)
M.B.A.	26 (8.2)	Sales achievement awards	
Company status		Yes	276 (86.8)
Multinational	93 (29.2)	No	42 (13.2)
National	222 (69.8)	Sales target	
Franchise	1 (0.3)	Easily achievable	99 (31.1)
Own	2 (0.6)	Difficult to achieve	203 (63.8)
Job title		Not achievable	16 (5.0)
Junior medical representative	271 (85.2)	Conveyance allowance	
Senior medical representative	35 (11.0)	Yes	167 (52.5)
1st line manager	11 (3.5)	No	151 (47.5)
2nd line manager	1 (0.3)	Manager behavior	
Monthly income (Pakistan Rupees)		Good	178 (56.0)
<15,000	14 (4.4)	Neutral	119 (37.4)
15,000–25,000	102 (32.1)	Bad	21 (6.6)
26,000–35,000	106 (33.3)	Doctor behavior	
36,000–50,000	48 (15.1)	Good	125 (39.3)
>50,000	48 (15.1)	Neutral	160 (50.3)
Job experience (years)		Bad	33 (10.4)
<1	76 (23.9)	Product sample available	
1–2	48 (15.1)	Yes	287 (90.3)
3–4	48 (15.1)	No	31 (9.7)
≥5	146 (45.9)	Call timing	
Job security		Manageable	177 (55.7)
Yes	121(38.1)	Difficult to manage	132 (41.5)
No	197 (61.9)	Not manageable	9 (2.8)
No. of calls/day		Specialty group	
<10	49 (15.4)	Yes	142 (44.7)
10–15	160 (50.3)	No	176 (55.2)
16–20	92 (28.9)	Pension/Gratuity/Provident fund	
20–25	10 (3.1)	Yes	200 (62.9)
>25	7 (2.2)	No	118 (37.1)
Insufficient family time			
Yes	179 (56.3)		
No	139 (43.7)		

*B.Sc.* Bachelor of Science, *B.A.* Bachelor of Arts, *B.Com.* Bachelor of Commerce, *M.B.A.* Master of Business Administration

### Mean PHQ-8 scores

The mean PHQ-8 score for the study population was 5.50 (SD = 5.15). According to the results obtained in this study, 52 (16.4 %) respondents had severe depression, whereas eight (2.5 %) respondents had severe major depression. The remaining majority (n = 258, 81.1 %) of respondents did not have clinical depression (Table 2). Distribution of mean (SD) PHQ-8 scores across patients’ characteristics is provided in the Additional file 1: Table S1.

**Table 2:** Distribution of PHQ-8 scores among the study participants

PHQ-8 scoring assumption	Depression level	Frequency (%)
<10	No clinical depression	258 (81.1)
10–19	Major depression	52 (16.4)
≥20	Severe major depression	8 (2.5)

### Predictors of depression

Table 3 shows the final predictors of depression among the study respondents. Through multivariate analysis, the factors that were associated with depression included insufficient time for the family (p = .025; AOR 2.138) and unsatisfactory behaviour of the managers (p = .044; AOR 1.999). This model fit was based on a non-significant Hosmer and Lemeshow test (p = 0.488).

**Table 3:** Final predictors of major and severe major depression: logistic regression analysis

Variables	B	SE	Sig.	AOR	95.0 % C.I. for Exp (B)
Insufficient family time	.760	0.338	*.025*	2.138	1.102, 4.148
Unsatisfactory doctor behaviour	.322	0.375	.391	1.380	0.662, 2.878
Unsatisfactory manager behaviour	.692	0.343	*.044*	1.999	1.020, 3.915
No sales achievement award	.738	0.414	.075	2.092	0.929, 4.711
No annual sales conference	.412	0.427	.335	1.509	0.653, 3.485
Monthly income less than 36,000 PKR	.604	0.379	.111	1.829	0.871, 3.842

p-value less than 0.05 in italic. *PKR* Pakistan Rupees, Model summary = Chi square (30.797), df (6), p < 0.0005; Nagelkerke R Square (.149); Hosmer and Lemeshow Chi square test (7.455), p = .488

### Mean SF-36v2 scores

The mean (SD) scores of eight health domains and summary components are shown in Table 4. The highest mean score was observed for the VT scale (53.7, SD = 9.85), whereas the lowest mean score was noted for the RE scale (37.62, SD = 10.09). The mean PCS and MCS scores were 48.59 (SD = 7.48) and 43.20 (SD = 9.94), respectively (Table 2). Distribution of mean (SD) HRQoL scores across patients’ characteristics is provided in the Additional file 1: Table S1.

**Table 4:** SF-36v2 norm-based scores of eight health domains and summary components scores using the standard scoring algorithms

Scales	Minimum score	Maximum score	Mean (SD)
PF	19.26	57.54	47.39 (8.98)
RP	21.23	57.16	40.37 (7.99)
BP	21.68	62.00	47.93 (10.01)
GH	18.95	66.50	51.13 (9.90)
VT	22.89	70.42	53.76 (9.85)
SF	17.23	57.34	41.45 (9.66)
RE	14.39	56.17	37.62 (10.09)
MH	11.63	63.95	45.92 (11.57)
PCS	19.56	66.26	48.59 (7.48)
MCS	8.97	62.82	43.20 (9.94)

SF-36v2 norm based scores of eight health domains and summary components scores along internal consistency of Urdu version of SF-36v^2^ health survey

Scales: *PF* physical functioning, *RP* role-physical, *RE* role-emotion, *BP* bodily pain, *VT* vitality, *SF* social functioning, *GH* general health, *MH* mental health, *PCS* physical component summery, *MCS* mental component summery

### Predictors of health-related quality of life

In multiple linear regression analysis, depression (p < .0005) and difficulty in achieving sales target (p = .048) were independently associated with lower PCS scores. Of the statistically significant variables, depression (β = −.256) made the largest unique contribution (Table 3). Similarly, depression (p < .0005), insufficient family time (p = .002) and monthly income less than 36,000 PKR (p = .004) were associated with lower MCS scores. Of these statistically significant variables, depression (β = −.356) made the largest unique contribution followed by monthly income less than 36,000 PKR (β = −.163) (Table 5).

**Table 5:** Final predictors of physical and mental component summary: multiple linear regression analysis

Variables	B	SE	Sig.	95.0 % C.I. for beta
Physical component summary*				
Depression	−.256	1.038	*<.0005*	−6.929, −2.844
Insufficient family time	−.102	0.813	.060	−3.139, 0.062
Sales target not achievable	−.106	0.861	*.048*	−3.401, −0.014
No sales achievement awards	−.088	1.290	.133	−4.483, 0.593
No annual sales conference	−.048	1.287	.398	−3.596, 1.434
No pension and gratuity	−.087	0.836	.107	−2.997, 0.292
Mental component summary^†^				
Depression	−.356	1.300	*<.0005*	−11.586, −6.468
Insufficient family time	−.159	1.025	*.002*	−5.193, −1.158
Sales target not achievable	−.072	1.089	.158	−3.684, 0.601
No sales achievement awards	.034	1.655	.553	−2.272, 4.239
No annual sales conference	−.096	1.625	.082	−6.031, 0.364
Unsatisfactory doctor behavior	−.031	1.025	.539	−2.647, 1.387
No conveyance allowance	−.016	1.096	.772	−2.474, 1.838
No annual leaves	−.022	1.417	.697	−3.341, 2.237
No professional qualification	−.023	1.184	.660	−2.851, 1.808
Monthly income less than 36,000 PKR	−.163	1.198	*.004*	−5.873, −1.158

p-value less than 0.05 in italic

*PKR* Pakistan Rupees

* Model summary: R^2^ = 0.146, p < 0.0005

^†^Model summary: R^2^ = 0.268, p < 0.0005

**Fig. 1:**
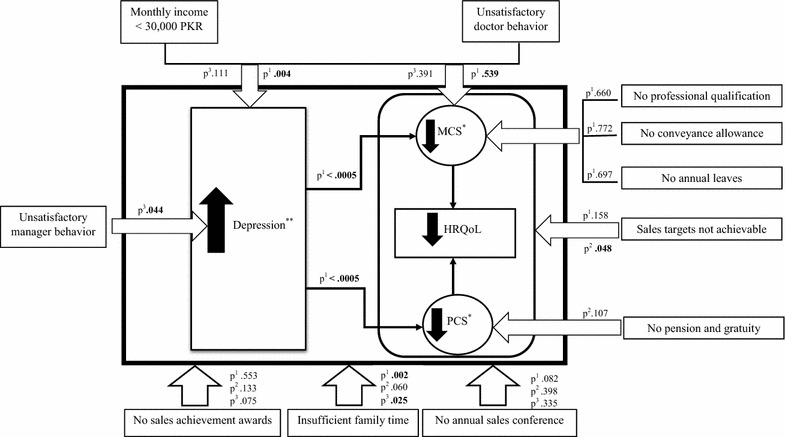
Summary of the regression analysis results relating health-related quality of life, depression and other study variables

Results from regression analysis are summarized in a model, relating HRQoL, depression and other study variables (Fig. 1).

## Discussion

The pharmaceutical market has undergone enormous transitions in Pakistan. In the late eighties, the Pakistani pharmaceutical market was under the strong influence of multinational companies. After half a century of evolutionary period, the ratio of national and multinational companies has shifted to 55 and 45 % respectively (Zaman 2011). So with growth, there has been some significant competition between national and multinational pharmaceutical firms. This has raised demands of using modern sales and marketing techniques to achieve targets including; field optimization, use of promotional tools (giveaways, samples) (Kuatbayeva 2013), internet oriented marketing, e-detailing using i-pads and use of sales analysis software (Raheem et al. 2014).

The current marketing strategy has exerted excessive pressure on the MSRs not only throughout the world in general, but also in Asia, specifically in South Asia. Pakistan is not exempt from this and our study suggests MSRs are under pressure through growing competition in the pharmaceutical marketplace. The pressure relates to escalating sales targets, directly affecting physical and emotional wellbeing and causing an elevation in levels of depression. This depression in MSRs can take any form such as, social isolation (Sadock and Sadock 2008), low self-esteem, low productivity and disrupted social and family life through disordered interpersonal relationships resulting in a poor HRQoL (Behrman and Perreault 1984).

Published studies confirm that the PHQ-8 is a valid diagnostic and severity measure for depressive disorders. These studies confirmed that a PHQ-8 score of ≥10 represents clinically significant depression (Kroenke et al. 2001, 2009). A study further confirmed that DSM-IV based diagnostic algorithm and PHQ-8 score ≥10 yielded similar results (Kroenke et al. 2009). Kroenke and co-workers stated that PHQ-8 is more convenient to use than the diagnostic algorithm (i.e., DSM-IV) (Kroenke et al. 2001). According to our findings, a greater number of respondents were suffering from clinical depression (major depression and severe major depression). This indicates that they had one or more of these symptoms; loss of interest in the normal daily activities, hopelessness, restlessness, altered sleep patterns and eating habits, fatigue, worthlessness, guilt, self-hate and persistent suicidal thoughts along with the symptoms of severe depression (Thase 2000). Previous studies from different parts of the world have also reported stress among the pharmaceutical sales force (Patil and Meena 2013; Harris et al. 2003; Tander et al. 2007). This fact is alarming and requires urgent attention by the appropriate authorities.

The study highlighted that the predictors of depression among the MSRs were insufficient time for the family and unsatisfactory behaviour of pharmaceutical company managers. Published studies have unveiled a reality that inadequate family time due to longer working hours cause mental distress. A positive association between work time and family conflict (Brenes 2007; Major et al. 2002) has also been made. In our study, a large proportion of the respondents were married, some with children and independently running their families. Inability to spend sufficient time with their families due to unpredictable working schedules, dealing with a large number of calls per day and ever changing meeting times with doctors became a major cause of depression among them. This has also been reported by Patil and Meena that long working hours, dissatisfaction with their job, continuous pressure to increase the sales and conflicting demands between work and home were some major causes of stress among the MSRs in their study in India (Patil and Meena 2013).

Looking at the second major predictor of depression in the respondents (i.e. unsatisfactory manager behaviour), various studies have shown that managers often behaved in an inappropriate manner toward their field force, posing extra pressure on their subordinates to get their sales targets achieved (Hales 1986). Unsatisfactory behaviour of the managers may lead to the development of low self-esteem and depression in the MSRs, hence disturbing their work and social lives. Furthermore, some studies declared that workplace bullying has a strong connection with depression (World Health Organization 2003). Bullying behaviour of managers towards MSRs may influence levels of depression and diminish their HRQoL.

The findings of the SF36-v2 Health Survey showed that the mean MCS scores (43.20 NBS) of our study participants was less than the general population norms. Low MCS scores was an indication of mental and psychological morbidity among the study participants. Based on the MCS scores, we can assume that the study participants had frequent psychological distress and they experienced social and role disability due to emotional problems. Within the mental health domain, the RE and SF scales were most affected. Low mean scores of the RE scale showed that our respondents had emotional stress and that was affecting their work and daily routine. Similarly, low mean scores of the SF scale showed that the MSRs had extreme and frequent interference with normal social activities due to physical and emotional problems (Ware et al. 2007). Excessive work load, inability to cope with the large number of calls every day and frustration owing to prolonged clinic waiting times are possible reasons for altered mental health of our study participants (Islam 2012). Similarly, limited opportunity to interact with the colleagues and friends may be another reason for frustration and mental stress among the MSRs (Harris et al. 2003). Mental morbidity is alarming as it could adversely affect the family and social life of this sub-group of people, leading to diminished working ability.

Interestingly, the physical health of our respondents was within the normal range of general population scores (47–53 NBS). Within the physical health domain, the higher VT scores showed that the respondents were full of energy. In Pakistan, the pharmaceutical companies employ young and energetic people in their sales and marketing teams. In this study, more than 85 % of respondents were less than 35 years of age which might be a reason for their better physical health.

Our study showed multiple predictors of poor HRQoL among the MSRs. The major predictor of diminished HRQoL was depression which was observed as the major cause of decline in both physical and mental health. A study conducted in the USA confirmed a direct relationship between depression and reduced physical activities (Goodwin 2003). Similarly, the World Health Organization report on mental health has confirmed that psychological and social factors lead to physical illness, mental and behavioral disorders which adversely affect the quality of life of an individual (World Health Organization 2001).

Sales forces are motivated and show high performance with achievable and reasonable sales targets. Unrealistically high sales targets not only reduce their performance but also effect HRQoL in a negative manner. In our study, it was observed that unachievable sales targets was a predictor of poor physical health among the MSRs. Physical stress due to difficulty in achieving sales targets is logical as in order to improve the sales performance, MSRs may have to face prolonged driving, longer waiting times outside physician’s clinics and manual handling of the promotional materials (Sang et al. 2010; Tander et al. 2007; Sakakibara et al. 2006).

Family is a core component of one’s life which provides a reason and energy to live a happy and healthy life. Conflicting demands between work and family may lead to mental stress among people. Our study also confirmed that inadequate family time precipitated poor mental health among the MSRs. Economic well-being is a hallmark of a good quality of life (Niedzwiedz et al. 2012; Cassedy et al. 2013; Pawlinska-Chmara et al. 2013; Burstrom et al. 2001; Ma and McGhee 2013). Low income results in lower social status and poorer lifestyle which contributes to an unhealthy life (Andrews and Withey 2012). In our study, lower MCS scores among the low income group of MSRs add weight to these arguments.

The findings of our study should not be generalized for the whole of Pakistan because the sample was drawn from only two districts of the Punjab province. Nevertheless, the sample may be representative to an extent because almost all of the pharmaceutical companies in Pakistan have deputed their sales and marketing staff in all major districts including Lahore and Bahawalpur. Lahore is the hub of pharmaceutical marketing and distribution and Bahawalpur is one of the major cities of Southern Punjab. As such, the sampling from these districts is sufficient to highlight the issues related to the MSRs working in Pakistan. We did not study the effect of moderator and mediator variables in our analysis model. Future studies may explore this in detail by employing techniques such as structural equation modelling (SEM).

## Conclusion

This study has confirmed the impaired mental health and prevalence of depression among the MSRs workforce in Pakistan. Compromised mental health and the prevalence of depression among the MSRs suggest pharmaceutical companies need to devise health management strategies and interventions to ensure effective prevention and management of mental health problems among Pakistani MSRs. Furthermore, the regulatory authorities working for the protection of labor rights in Pakistan should also formulate such policies so as to protect the rights of the MSRs and to ensure their mental and physical wellbeing. In this respect, special attention should be given to these identified high risk groups.

## Additional file

10.1186/s40064-016-2716-1 Distribution of mean SF-36v2 and PHQ-8 scores across the study variables.

## Abbreviations

AOR: Adjusted odds ratio
BP: Bodily pain
DSM-IV: Diagnostic and Statistical Manual of Mental Disorders-4th Edition
GH: General health
HRQoL: Health-related quality of life
MCS: Mental component summery
MH: Mental health
MSRs: Medical sales representatives
NBS: Norm based scores
PCS: Physical component summery
PHQ: Personal Health Questionnaire
PF: Physical functioning
RE: Role-emotion
RP: Role-physical
SF: Social functioning
SF-36v2: Short form-36v2
VT: Vitality
