# Supplementary material for: Health-related quality of life and depression among medical sales representatives in Pakistan
Source: Springerplus. 2016 Jul 11;5(1):1048. doi: 10.1186/s40064-016-2716-1 (PMC4940353; doi:10.1186/s40064-016-2716-1)
Supplement: Supplementary file 1 — 10.1186/s40064-016-2716-1 Distribution of mean SF-36v2 and PHQ-8 scores across the study variables. [file 40064_2016_2716_MOESM1_ESM.docx]

# Health-Related Quality of Life and Depression among Medical Sales Representatives in Pakistan

Muhammad Atif ^1^, Arslan Bashir ^1*^, Quratulain Saleem ^1^, Rabia Hussain ^2^, Shane Scahill ^3^, Zaheer-Ud-Din Babar ^4^

^1^ Department of Pharmacy, The Islamia University of Bahawalpur, Punjab, Pakistan

^2^ Lahore Pharmacy College, Lahore Medical and Dental College, Pakistan

^3^ School of Management, Massey University, New Zealand

^4^ School of Pharmacy, University of Auckland, New Zealand

**Journal:** SpringerPlus

**^*^Corresponding Author**

**Arslan Bashir**

Department of Pharmacy, The Islamia University of Bahawalpur, Punjab, Pakistan

Email: arslanbashir41@gmail.com, Phone: +923448205957

| **Characteristics** | **SF-36v2 scores**  **Mean (SD)** | | **PHQ-8 scores**  **Mean (SD)** | **Characteristics** | **SF-36v2 scores**  **Mean (SD)** | | **PHQ-8 scores**  **Mean (SD)** |
| --- | --- | --- | --- | --- | --- | --- | --- |
|  | **PCS** | **MCS** |  |  | **PCS** | **MCS** |  |
| ***Gender*** |  |  |  | ***Quarterly shopping cards*** |  |  |  |
| Male | 48.66 (7.31) | 43.26 (9.94) | 5.50±5.16 | Yes | 49.11±7.32 | 43.39±9.57 | 4.14±4.96 |
| Female | 45.99 (12.42) | 41.32 (10.32) | 5.56±5.15 | No | 48.54±7.51 | 43.18±9.99 | 5.63±5.15 |
| ***Age group (years)*** |  |  |  | ***Annual leaves*** |  |  |  |
| 18-24 | 49.16 (6.94) | 43.16 (10.20) | 5.68±3.84 | Yes | 48.88±7.42 | 43.79±9.73 | 5.26±5.12 |
| 25-34 | 48.86 (7.43) | 43.27 (9.68) | 5.38±5.12 | No | 47.35±7.68 | 40.71±10.49 | 6.52±5.17 |
| 35-44 | 47.64 (7.31) | 44.17 (10.43) | 5.29±5.89 | ***Periphery visits*** |  |  |  |
| 45-54 | 32.32 (2.89) | 25.35 (4.74) | 15.33±6.43 | Yes | 48.77±7.37 | 43.31±9.98 | 5.32±4.99 |
| ***Marital status*** |  |  |  | No | 48.08±7.81 | 42.90±9.87 | 6.01±5.56 |
| Single | 48.38 (7.12) | 42.33 (10.50) | 5.59±5.31 | ***Professional trainings*** |  |  |  |
| Married | 48.42 (7.64) | 43.23 (9.66) | 5.61±5.16 | Yes | 48.64±7.47 | 43.35±9.76 | 5.37±5.03 |
| Married with children | 52.04 (7.60) | 38.76 (9.02) | 7.50±9.19 | No | 48.18±7.65 | 42.13±11.27 | 6.50±5.94 |
| Divorced/separated | 49.78 (7.91) | 45.87 (9.48) | 4.61±4.49 | ***Annual sales conference*** |  |  |  |
| ***Title of last degree*** |  |  |  | Yes | 48.99±7.50 | 43.88±9.53 | 5.06±4.72 |
| BSc | 47.24 (6.69) | 43.11 (9.13) | 5.30±4.82 | No | 45.87±6.82 | 38.63±11.47 | 8.49±6.76 |
| BA/B.com | 48.70 (7.66) | 42.09 (10.26) | 5.86±5.17 | ***Sales target assigned*** |  |  |  |
| Masters | 48.20 (8.39) | 42.40 (11.09) | 7.15**±**5.84 | Yes | 48.75±7.28 | 43.20±9.95 | 5.42**±**5.09 |
| Pharmacy | 50.35 (6.84) | 46.30 (9.73) | 4.44±5.05 | No | 46.07±10.08 | 43.23±10.02 | 6.84±6.00 |
| MBA | 48.78 (8.40) | 44.00 (8.58) | 4.19±4.70 | ***Sales achievement awards*** | | | |
| ***Company status*** |  |  |  | Yes | 49.16±7.12 | 43.71±9.89 | 5.09±4.79 |
| Multinational | 48.53 (8.30) | 44.17 (10.11) | 5.81±5.90 | No | 44.84±8.74 | 39.89±9.74 | 8.24±6.50 |
| National | 48.49 (7.10) | 42.83±9.93) | 5.41±4.83 | ***Sales target status*** |  |  |  |
| Franchise | 61.15 (00.00) | 38.93 (00.00) | 6.00±0.00 | Easily achievable | 50.21±7.49 | 44.86±9.75 | 4.39±4.87 |
| Own | 55.41 (4.67) | 41.93 (3.01) | 1.50±2.12 | Difficult to achieve | 47.97±7.41 | 43.11±9.66 | 5.71±4.99 |
| ***Job title*** |  |  |  | Not achievable | 46.29±6.93 | 34.10±10.25 | 9.69±6.51 |
| Junior MSR | 48.81 (7.26) | 43.24 (9.84) | 5.53±4.97 | ***Conveyance allowance*** |  |  |  |
| Senior MSR | 47.46 (7.48) | 42.38 (10.50) | 4.97±6.25 | Yes | 48.86±7.92 | 44.51±10.04 | 5.04±5.17 |
| 1^st^ line manager | 46.39 (12.32) | 43.97 (11.49) | 7.00±5.85 | No | 48.29±6.98 | 41.76±9.66 | 6.02±5.09 |
| 2^nd^ line manager | 50.72 (0.0) | 52.48 (0.0) | 1.00±0.0 | ***Managers behavior*** |  |  |  |
| ***Monthly income (Pakistan Rupees)*** | | | | Good | 49.14±7.40 | 43.86±9.12 | 4.44±4.26 |
| <15,000 | 47.14 (5.94) | 38.66 (9.71) | 6.50±5.45 | Neutral | 48.16±7.62 | 43.00±10.73 | 6.42±5.81 |
| 15,000-25,000 | 47.39 (7.56) | 41.72 (11.21) | 6.54±5.61 | Bad | 46.30±7.08 | 38.78±11.22 | 9.29±5.49 |
| 26,000-35,000 | 49.79 (7.20) | 41.82 (9.04) | 5.14±4.69 | ***Doctors behavior*** |  |  |  |
| 36,000-50,000 | 47.49 (8.52) | 47.66 (8.57) | 5.46±5.38 | Good | 49.04±8.44 | 44.66±9.01 | 4.08±4.24 |
| >50,000 | 49.98 (6.80) | 46.27 (8.32) | 3.85±4.38 | Neutral | 48.36±6.63 | 43.04±10.07 | 6.20±5.46 |
| ***Job experience (year)*** |  |  |  | Bad | 47.95±7.65 | 38.48±11.37 | 7.52±5.47 |
| <1 | 47.00 (7.63) | 42.11 (9.44) | 4.83±4.68 | ***Product sample available*** |  |  |  |
| 1-2 | 48.46 (7.47) | 42.77 (11.39) | 6.79±5.48 | Yes | 48.85±7.25 | 43.34±10.06 | 5.34±5.05 |
| 3-4 | 49.08 (7.00) | 43.00 (10.10) | 5.77±4.30 | No | 46.13±9.14 | 41.91±8.84 | 7.00±5.82 |
| ≥5 | 49.29 (7.51) | 43.98 (9.67) | 5.34±5.48 | ***Call timing*** |  |  |  |
| ***Job Security*** |  |  |  | Manageable | 49.13±7.58 | 44.75±9.60 | 4.86±4.65 |
| Yes | 49.28 (8.05) | 44.47 (10.20) | 4.66±4.84 | Difficult to manage | 48.02±7.38 | 41.48±9.91 | 6.36±5.74 |
| No | 48.16 (7.10) | 42.43 (9.72) | 6.02±5.27 | Not manageable | 46.14±6.65 | 38.03±12.18 | 5.56±3.75 |
| ***No. of calls/day*** |  |  |  | ***Specialty group*** |  |  |  |
| <10 | 48.06 (8.88) | 43.26 (10.36) | 4.98±5.68 | Yes | 48.58±7.12 | 43.50±9.42 | 5.43±4.97 |
| 10-15 | 49.20 (7.39) | 44.12 (10.21) | 5.42±5.20 | No | 48.60±7.93 | 42.83±10.57 | 5.59±5.38 |
| 16-20 | 47.78 (7.04) | 41.87 (9.15) | 5.82±4.78 | ***Pension/Gratuity/Provident fund*** | | |  |
| 20-25 | 50.24 (6.66) | 42.07 (10.04) | 6.00±5.03 | Yes | 49.28±7.22 | 43.29±9.95 | 5.47±5.15 |
| >25 | 46.43 (5.50) | 40.93 (11.04) | 6.29±6.02 | No | 47.41±7.79 | 43.06±9.97 | 5.57±5.17 |
| ***Enough time for the family*** |  |  |  |  |  |  |  |
| Yes | 50.11 (6.59) | 46.39 (8.87) | 4.08±3.88 |  |  |  |  |
| No | 47.41 (7.93) | 40.80 (10.09) | 6.61±5.72 |  |  |  |  |

**Table S1:** Distribution of mean SF-36v2 and PHQ-8 scores across the study variables
